# Innovative rescue therapy for ulcer bleeding after band ligation: New ENDOLOOP/clips/grasping forceps technique

**DOI:** 10.1007/s12664-021-01177-5

**Published:** 2021-04-19

**Authors:** Hiroyuki Hisada, Akihiro Miyakawa, Kenji Shimura

**Affiliations:** 1 Department of Gastroenterology, The University of Tokyo, 7-3-1, Hongo, Bunkyo-ku, Tokyo, 113-8655 Japan; 2 Department of Gastroenterology, Asahi General Hospital, Chiba, Japan

**Fig. 1:**
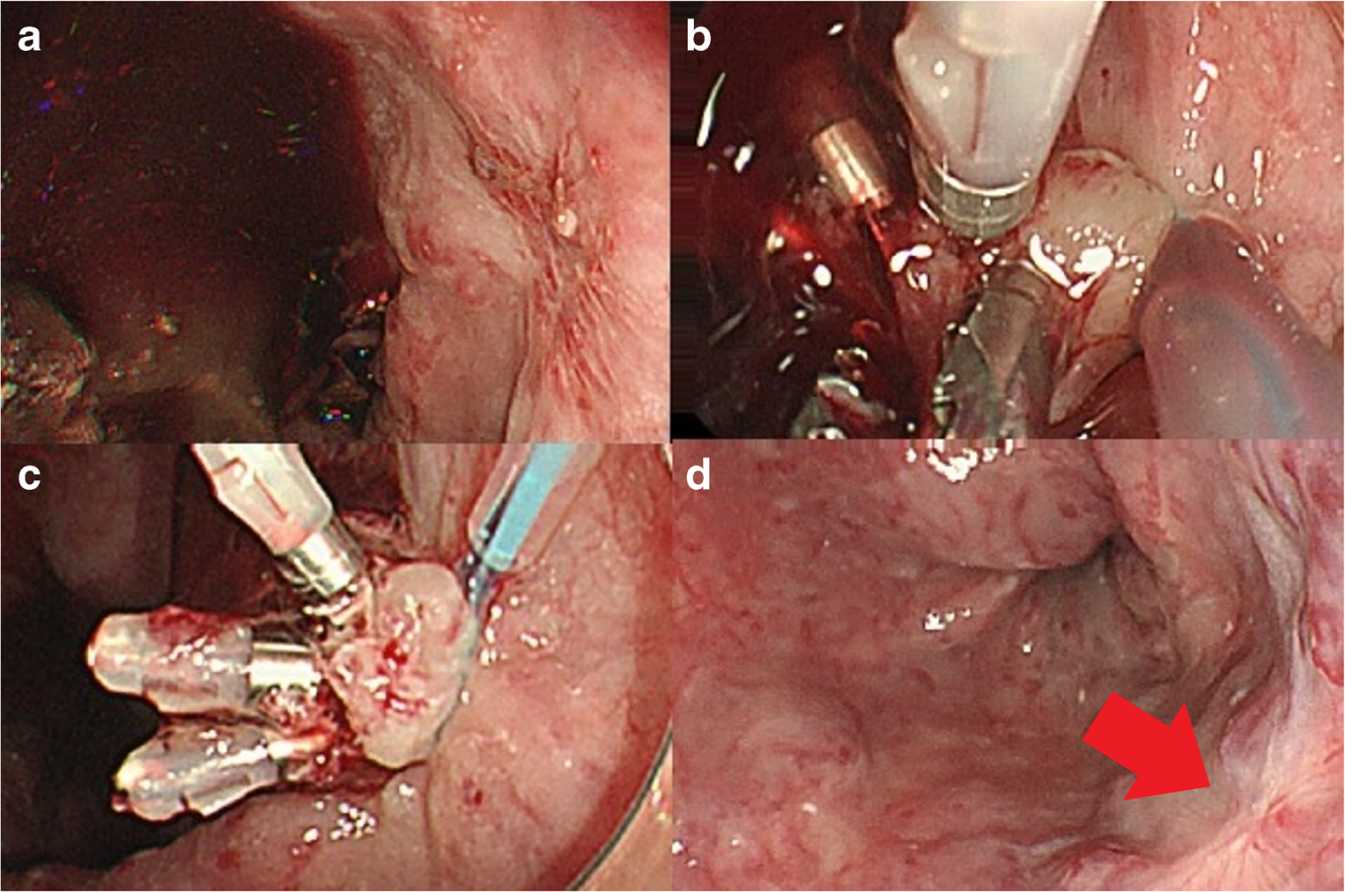
**a** Esophagogastroduodenoscopy showing a visibly bleeding vessel at the base of the band of ulcers. **b** After clipping of the bleeding vessel, grasping forceps were used to cover the bleeding vessel with mucosa, and the ENDOLOOP was then advanced to the base of the lesion. **c** The ENDOLOOP was placed at the base of the lesion. **d** There was no evidence of new bleeding at the 3-month follow-up

A 72-year-old man presented with chronic hepatitis C infection and esophageal variceal bleeding. He had undergone endoscopic variceal ligation (EVL) 4 months earlier for hemostatic control. Esophagogastroduodenoscopy revealed a band ulcer with a visibly bleeding vessel (Fig. 1a). On repeat EVL, hemostasis could not be achieved because of decreased tissue compliance. A Sengstaken–Blakemore (SB) tube (Sumitomo Bakelite Co., Ltd., Tokyo, Japan) was placed as a temporary measure. A second endoscopic procedure was performed the next day to remove the SB tube; rebleeding was observed. Instead of performing EVL again, we placed three endoclips near the bleeding vessel. The ENDOLOOP was then placed over the endoclips, near the base of the mucosa, to close the vessel in a purse-string manner by using a two-channel endoscope; the grasping forceps were used to cover the vessel with mucosa. Successful hemostasis was achieved (Fig. 1b, c); there was no evidence of new bleeding 3 months after surgery (Fig. 1d).

Esophageal variceal bleeding is one of the most common complications of liver cirrhosis. EVL is a widely accepted treatment of bleeding and is superior to endoscopic sclerotherapy as it involves fewer complications. EVL-induced ulcer formation is a known serious complication of this procedure, with a mortality rate of 27.3% [1]; no clear treatment has been described for it yet. Herein, we describe our approach to successful hemostasis of an EVL-induced ulcer with the use of a two-channel endoscope (GIF-2TQ260M, Olympus, Tokyo, Japan), grasping forceps (FG-42L-1, Olympus, Tokyo, Japan), endoclips (EZ clip, Olympus, Tokyo, Japan), and an ENDOLOOP (Polyloop; HX-400U-30, Olympus, Tokyo, Japan).

We placed the endoscopic clipping to form an anchor and then placed the ENDOLOOP at the base of the mucosa using grasping forceps that were used to cover the bleeding vessel with mucosa. Our novel approach helped achieve hemostatic control of bleeding of EVL-induced ulcer and should be considered a new treatment modality to control initial bleeding.

The prevalence of EVL-induced ulcer bleeding is only 3.6%, but it is fatal in some cases [1]. Several treatment methods, including endoscopic injection sclerotherapy, EVL, transjugular intrahepatic portosystemic shunt, over-the-scope clipping [2], and self-expanding metal stent [3], have been suggested; however, the methods used here may be safer and more economical than these modalities. Moreover, this method has been used for hemostasis of postpolypectomy bleeding and nonvariceal upper gastrointestinal bleeding [4, 5].

We believe that the combined use of endoclips with an ENDOLOOP is effective and economical in the management of EVL-induced ulcers.
